# Bis(2-amino-4-methyl­pyridinium) *trans*-diaqua­bis­(pyrazine-2,3-dicarboxyl­ato)cuprate(II) hexa­hydrate

**DOI:** 10.1107/S1600536810023081

**Published:** 2010-06-23

**Authors:** Hossein Eshtiagh-Hosseini, Fabienne Gschwind, Nafiseh Alfi, Masoud Mirzaei

**Affiliations:** a Department of Chemistry, School of Sciences, Ferdowsi University of Mashhad, Mashhad 917791436, Iran; b Department of Chemistry, University of Fribourg, Chemin Du Musée 9, 1700 Fribourg, Switzerland

## Abstract

The title compound, (C_6_H_9_N_2_)_2_[Cu(C_6_H_2_N_2_O_4_)_2_(H_2_O)_2_]·6H_2_O, consists of a mononuclear *trans*-[Cu(pzdc)_2_(H_2_O)_2_]^2−^ dianion (pzdc is pyrazine-2,3-dicarboxyl­ate) and two [ampyH]^+^ cations (ampy is 2-amino-4-methyl­pyridine) with six water mol­ecules of solvation. The Cu^II^ atom is hexa­coordinated by two pzdc groups and two water mol­ecules. The coordinated water mol­ecules are in *trans*-diaxial positions and the pzdc dianion acts as a bidentate ligand through an O atom of the carboxyl­ate group and the N atom of the pyrazine ring. There are diverse hydrogen-bonding inter­actions, such as N—H⋯O and O—H⋯O contacts, which lead to the formation of a three-dimensional supra­molecular architecture.

## Related literature

For the crystal structure of pyrazine-2,3-dicarb­oxy­lic acid (pzdcH_2_), see: Takusagawa & Shimada (1973 ▶). For complexes of pzdcH_2_ with manganese and zinc, see: Eshtiagh-Hosseini *et al.* (2010*a* ▶,*b* ▶). For the structure of bis­(2,4,6-triamino-1,3,5-triazin-1-ium) pyrazine-2,3-dicarboxyl­ate tetra­hydrate, see: Eshtiagh-Hosseini *et al.* (2010*c* ▶). For a review articleon water cluster chemistry, see: Aghabozorg *et al.* (2010 ▶).
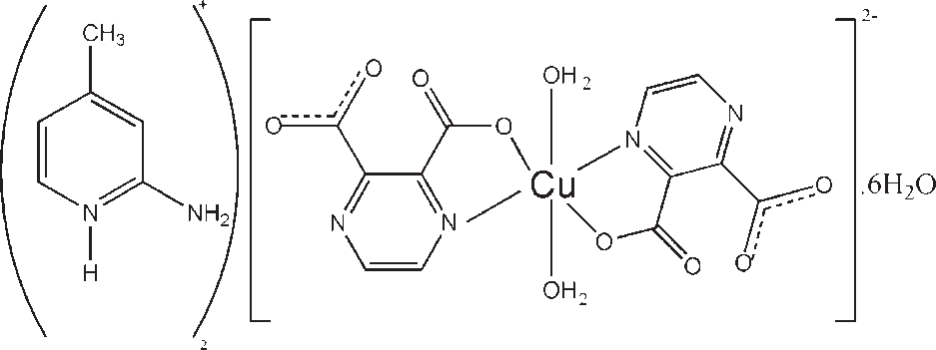


## Experimental

### 

#### Crystal data

(C_6_H_9_N_2_)_2_[Cu(C_6_H_2_N_2_O_4_)_2_(H_2_O)_2_]·6H_2_O
*M* *_r_* = 758.17
Triclinic, 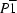

*a* = 6.9075 (14) Å
*b* = 8.4710 (17) Å
*c* = 14.505 (3) Å
α = 78.28 (3)°
β = 83.62 (3)°
γ = 85.81 (3)°
*V* = 824.8 (3) Å^3^
*Z* = 1
Mo *K*α radiation
μ = 0.75 mm^−1^
*T* = 293 K
0.3 × 0.2 × 0.1 mm

#### Data collection

Stoe IPDS 2 diffractometer
Absorption correction: for a sphere [modified Dwiggins (1975 ▶) interpolation procedure] *T*_min_ = 0.743, *T*_max_ = 0.745
17015 measured reflections
4684 independent reflections
4282 reflections with *I* > 2σ(*I*)
*R*_int_ = 0.046

#### Refinement

*R*[*F* ^2^ > 2σ(*F* ^2^)] = 0.036
*wR*(*F* ^2^) = 0.092
*S* = 1.04
4684 reflections
268 parameters
H atoms treated by a mixture of independent and constrained refinement
Δρ_max_ = 0.36 e Å^−3^
Δρ_min_ = −0.52 e Å^−3^

### 

Data collection: *X-AREA* (Stoe & Cie, 2009 ▶); cell refinement: *X-RED* (Stoe & Cie, 2009 ▶); data reduction: *X-RED*; program(s) used to solve structure: *SHELXS97* (Sheldrick, 2008 ▶); program(s) used to refine structure: *SHELXL97* (Sheldrick, 2008 ▶); molecular graphics: *DIAMOND* (Crystal Impact, 2009 ▶); software used to prepare material for publication: *publCIF* (Westrip, 2010 ▶).

## Supplementary Material

Crystal structure: contains datablocks I, global. DOI: 10.1107/S1600536810023081/fj2310sup1.cif

Structure factors: contains datablocks I. DOI: 10.1107/S1600536810023081/fj2310Isup2.hkl

Additional supplementary materials:  crystallographic information; 3D view; checkCIF report

## Figures and Tables

**Table 1:** Hydrogen-bond geometry (Å, °)

*D*—H⋯*A*	*D*—H	H⋯*A*	*D*⋯*A*	*D*—H⋯*A*
N3—H13⋯O4^i^	0.82	1.91	2.7221 (19)	169
N4—H14*A*⋯O8^ii^	0.77	2.12	2.8879 (19)	177
N4—H14*B*⋯O3^i^	0.84 (2)	2.07 (2)	2.903 (2)	168 (2)
O5—H5*B*⋯O7^i^	0.79 (3)	1.92 (3)	2.703 (2)	173 (3)
O5—H5*A*⋯O4^i^	0.82 (3)	2.09 (3)	2.8556 (18)	157 (3)
O8—H8*B*⋯O1^iii^	0.76 (3)	2.03 (3)	2.7838 (18)	173 (3)
O6—H6*B*⋯O4^iii^	0.81 (4)	2.06 (4)	2.839 (2)	162 (3)
O7—H17*B*⋯O8^iv^	0.76 (4)	2.04 (4)	2.797 (2)	172 (4)

Symmetry codes: (i) 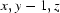; (ii) 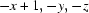; (iii) 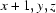; (iv) 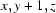.

**Fig. 1.**
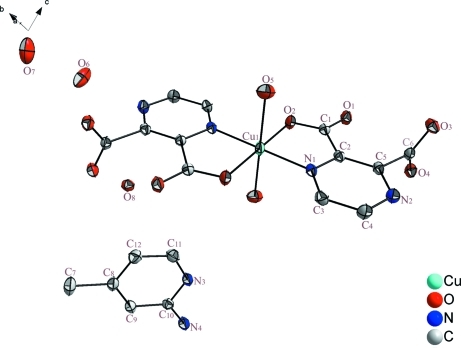
Molecular structure of (ampy)2[Cu(pzdc)2(H2O)2].6H2O. Ellipsoids are drawn at the 50% probability level. Hydrogen atoms are omitted for further clarity.

**Fig. 2.**
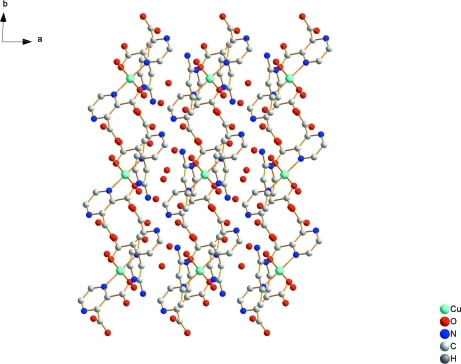
Packing diagram of (ampy)2[Cu(pzdc)2(H2O)2].6H2O.

## supplementary crystallographic information

### Comment

Dicarboxylate ligands are widely used to assemble supramolecular network organized by coordination bonds, hydrogen bonds and π –π stacking interaction. Due to the manifold N- and O-donors of pyridine or pyrazine-(di)carboxylic ligands, metal pyridine- or pyrazine dicarboxylates can contrast versatile structural motifs, which finally aggregate to generate various supramolecular architectures with interesting properties. As ones of the dicarboxylate ligands, pzdcH_2_ have drawn extensive attentions. For the first time, Takusagawa & Shimada (1973) by single crystal X-ray diffraction, determined the structure of pzdcH_2_. Continuing with our previous works on synthesizing coordination compounds *via* proton transfer mechanism including zinc atom (Eshtiagh-Hosseini *et al.*, 2010a), manganese atom (Eshtiagh-Hosseini *et al.*, 2010b), Bis(2,4,6-triamino-1,3,5-triazin-1-ium) pyrazine-2,3-dicarboxylate tetrahydrate (Eshtiagh-Hosseini *et al.*, 2010c), herein, we planned the reaction between pzdcH_2_, ampy, and copper(II) choloride which resulted in the formation of (ampy)_2_[Cu(pzdc)_2_(H_2_O)_2_]. 6H_2_O (Fig. 1). Crystal packing diagram related to the title compound is also rendered in the Fig. 2. As you can see, the equatorial plane is occupied by two (pzdc)^2-^ ligands coordinating through the pyridine nitrogen and one oxygen of the deprotonated carboxylate groups. The two coordinated water molecules occupy axial plane. This compound consists of an anionic moiety, *trans*-[Cu(pzdc)_2_(H_2_O)_2_]^2-^ complex, counter-ions, (ampy)^+^, and six uncoordinated water molecules. The Cu—O and Cu—N bond distances related to (pzdc)^2-^ ligand in herein presented compound are in the category of 1.9644 (13) Å , and 1.9840 (14) Å, respectively. These observerd bond lenghts are comprable with Zn(II) polymeric coordination compound which recently reported by our research group (Eshtiagh-Hosseini *et al.*, 2010a ). In this polymeric compound which consist of only (pzdc)^2-^ coordinative ligand, {(C_3_H_12_N_2_)_2_[Zn(C_10_H_2_O_8_)_2_]}_n_, Zn—O and Zn—N bond distances are 2.0317 (15) to 2.2437 (15) Å, and 2.0901 (18) Å, respectively. These data show in this polymeric compound Zn—O bond distance is longer than herein presented compound. The intermolecular forces between the anionic and cationic parts in the title compound consist of hydrogen bonding and ion pairing interactions. Indeed, six uncoordinated water molecules increase the number of hydrogen bonds in the crystalline network and lead to the formation of (H_2_O)_n _clusters throughout the crystalline network (see Review article by Aghabozorg *et al.* 2010).

### Experimental

A solution of pzdcH_2_ (0.18 mmol, 0.03 mg) in water (10 ml) refluxed for 1 hr, then a solution of CuCl_2_.6H_2_O (0.02 mmol, 0.01 g) was added dropwise and continued refluxing for 6 hrs at 60°C. The obtained blue solution gave blue block like crystals of title compound after slow evaporation of solvent at room temperatur.

### Refinement

The space group was confirmed by using PLATON software package. The structure was solved by direct methods using SHELXS-97 and refined using full matrix least-squares on *F*^2 ^with the SHELX-97 package. H-Atoms were constrained to the parent site using a rigid model. A final verification of possible voids was performed using the VOID routine on PLATON software.

### Crystal data

**Table:**

(C_6_H_9_N_2_)_2_[Cu(C_6_H_2_N_2_O_4_)_2_(H_2_O)_2_]·6H_2_O	*Z* = 1
*M**_r_* = 758.17	*F*(000) = 395.0
Triclinic, *P*1	*D*_x_ = 1.526 Mg m^−^^3^
Hall symbol: -P 1	Mo *K*α radiation, λ = 0.71073 Å
*a* = 6.9075 (14) Å	Cell parameters from 34097 reflections
*b* = 8.4710 (17) Å	θ = 3,8–59,7°
*c* = 14.505 (3) Å	µ = 0.75 mm^−^^1^
α = 78.28 (3)°	*T* = 293 K
β = 83.62 (3)°	Block, blue
γ = 85.81 (3)°	0.3 × 0.2 × 0.1 mm
*V* = 824.8 (3) Å^3^	

### Data collection

**Table:**

Stoe IPDS 2 diffractometer	4684 independent reflections
Radiation source: fine-focus sealed tube	4282 reflections with *I* > 2σ(*I*)
graphite	*R*_int_ = 0.046
Detector resolution: 6.67 pixels mm^-1^	θ_max_ = 29.8°, θ_min_ = 2.5°
rotation method scans	*h* = −9→9
Absorption correction: for a sphere modified Dwiggins (1975) interpolation procedure	*k* = −11→11
*T*_min_ = 0.743, *T*_max_ = 0.745	*l* = −20→20
17015 measured reflections	

### Refinement

**Table:**

Refinement on *F*^2^	Primary atom site location: structure-invariant direct methods
Least-squares matrix: full	Secondary atom site location: difference Fourier map
*R*[*F*^2^ > 2σ(*F*^2^)] = 0.036	Hydrogen site location: inferred from neighbouring sites
*wR*(*F*^2^) = 0.092	H atoms treated by a mixture of independent and constrained refinement
*S* = 1.03	*w* = 1/[σ^2^(*F*_o_^2^) + (0.0453*P*)^2^ + 0.5453*P*] where *P* = (*F*_o_^2^ + 2*F*_c_^2^)/3
4684 reflections	(Δ/σ)_max_ = 0.001
268 parameters	Δρ_max_ = 0.36 e Å^−^^3^
0 restraints	Δρ_min_ = −0.52 e Å^−^^3^

### Special details

**Table:**

Experimental. Absorption correction: Interpolation using Int. Tab. Vol. C (1992) p. 523, Tab. 6.3.3.3 for values of muR in the range 0-2.5, and Int. Tab. Vol. II (1959) p. 302; Table 5.3.6 B for muR in the range 2.6-10.0. The interpolation procedure of C. W. Dwiggins Jr is used with some modification.
Geometry. All s.u.'s (except the s.u. in the dihedral angle between two l.s. planes) are estimated using the full covariance matrix. The cell s.u.'s are taken into account individually in the estimation of s.u.'s in distances, angles and torsion angles; correlations between s.u.'s in cell parameters are only used when they are defined by crystal symmetry. An approximate (isotropic) treatment of cell s.u.'s is used for estimating s.u.'s involving l.s. planes.
Refinement. Refinement of *F*^2^ against ALL reflections. The weighted *R*-factor wR and goodness of fit *S* are based on *F*^2^, conventional *R*-factors *R* are based on *F*, with *F* set to zero for negative *F*^2^. The threshold expression of *F*^2^ > 2σ(*F*^2^) is used only for calculating *R*-factors(gt) etc. and is not relevant to the choice of reflections for refinement. *R*-factors based on *F*^2^ are statistically about twice as large as those based on *F*, and *R*- factors based on ALL data will be even larger.

### Fractional atomic coordinates and isotropic or equivalent isotropic displacement parameters (Å^2^)

**Table:**

	*x*	*y*	*z*	*U*_iso_*/*U*_eq_	
Cu1	0.0000	0.0000	0.5000	0.01658 (8)	
O3	0.30782 (17)	0.49841 (13)	0.17617 (8)	0.0195 (2)	
O4	0.11843 (16)	0.64677 (13)	0.26546 (8)	0.0180 (2)	
O1	−0.09643 (16)	0.33706 (13)	0.27415 (8)	0.0190 (2)	
O5	0.1806 (2)	−0.17345 (17)	0.40519 (9)	0.0258 (3)	
O2	−0.12649 (16)	0.11778 (13)	0.38957 (8)	0.0189 (2)	
N1	0.19550 (18)	0.16670 (15)	0.45776 (8)	0.0147 (2)	
N2	0.43093 (18)	0.41844 (16)	0.37769 (9)	0.0173 (2)	
C1	−0.0415 (2)	0.24286 (17)	0.34432 (10)	0.0144 (2)	
C2	0.1458 (2)	0.27423 (16)	0.38093 (9)	0.0134 (2)	
C5	0.2651 (2)	0.40007 (17)	0.34084 (10)	0.0139 (2)	
C4	0.4760 (2)	0.31081 (19)	0.45398 (11)	0.0187 (3)	
H4	0.5897	0.3212	0.4806	0.022*	
C3	0.3583 (2)	0.18338 (18)	0.49501 (10)	0.0171 (3)	
H3	0.3936	0.1103	0.5483	0.021*	
C6	0.2244 (2)	0.52402 (17)	0.25287 (10)	0.0148 (2)	
N3	0.17729 (19)	−0.08982 (15)	0.12469 (9)	0.0175 (2)	
H13	0.1634	−0.1765	0.1614	0.021*	
N4	0.2985 (2)	−0.23942 (16)	0.01280 (10)	0.0200 (3)	
H14A	0.3217	−0.2450	−0.0394	0.024*	
C10	0.2467 (2)	−0.09571 (18)	0.03481 (10)	0.0160 (3)	
C9	0.2613 (2)	0.05143 (19)	−0.03132 (11)	0.0184 (3)	
C8	0.2056 (2)	0.19545 (18)	−0.00370 (11)	0.0197 (3)	
C11	0.1206 (2)	0.05082 (19)	0.15352 (11)	0.0212 (3)	
H11	0.0728	0.0489	0.2162	0.025*	
C12	0.1332 (3)	0.19396 (19)	0.09169 (12)	0.0223 (3)	
C7	0.2186 (3)	0.3528 (2)	−0.07308 (14)	0.0288 (4)	
H7A	0.2698	0.4314	−0.0443	0.043*	
H7B	0.0911	0.3898	−0.0912	0.043*	
H7C	0.3034	0.3379	−0.1281	0.043*	
O6	0.7121 (2)	0.67499 (18)	0.32078 (13)	0.0381 (4)	
O7	0.4817 (2)	0.9530 (2)	0.28417 (12)	0.0385 (3)	
O8	0.60159 (18)	0.25375 (14)	0.18411 (8)	0.0203 (2)	
H12	0.097 (4)	0.287 (3)	0.1154 (19)	0.040 (7)*	
H14B	0.289 (3)	−0.322 (3)	0.0565 (17)	0.024 (5)*	
H9	0.311 (3)	0.045 (2)	−0.0939 (15)	0.017 (5)*	
H5B	0.267 (4)	−0.130 (3)	0.372 (2)	0.040 (7)*	
H5A	0.138 (4)	−0.236 (3)	0.377 (2)	0.040 (7)*	
H8A	0.516 (4)	0.326 (3)	0.1824 (19)	0.039 (7)*	
H8B	0.677 (4)	0.279 (3)	0.2116 (19)	0.034 (6)*	
H6B	0.823 (5)	0.645 (4)	0.306 (2)	0.057 (9)*	
H6A	0.632 (6)	0.606 (5)	0.333 (3)	0.076 (11)*	
H17A	0.570 (5)	0.885 (4)	0.288 (2)	0.049 (8)*	
H17B	0.524 (5)	1.032 (4)	0.259 (3)	0.062 (10)*	

### Atomic displacement parameters (Å^2^)

**Table:**

	*U*^11^	*U*^22^	*U*^33^	*U*^12^	*U*^13^	*U*^23^
Cu1	0.01841 (13)	0.01479 (13)	0.01464 (12)	−0.00519 (9)	−0.00398 (9)	0.00436 (9)
O3	0.0237 (5)	0.0173 (5)	0.0150 (5)	0.0014 (4)	0.0007 (4)	0.0003 (4)
O4	0.0203 (5)	0.0131 (5)	0.0190 (5)	0.0006 (4)	−0.0017 (4)	−0.0002 (4)
O1	0.0208 (5)	0.0176 (5)	0.0172 (5)	−0.0032 (4)	−0.0062 (4)	0.0032 (4)
O5	0.0265 (6)	0.0295 (6)	0.0239 (6)	−0.0017 (5)	−0.0016 (5)	−0.0116 (5)
O2	0.0193 (5)	0.0175 (5)	0.0185 (5)	−0.0068 (4)	−0.0056 (4)	0.0037 (4)
N1	0.0172 (5)	0.0131 (5)	0.0129 (5)	−0.0012 (4)	−0.0023 (4)	0.0003 (4)
N2	0.0158 (5)	0.0174 (6)	0.0179 (6)	−0.0020 (4)	−0.0023 (4)	−0.0007 (5)
C1	0.0154 (6)	0.0140 (6)	0.0134 (6)	−0.0015 (5)	−0.0013 (5)	−0.0014 (5)
C2	0.0149 (6)	0.0124 (6)	0.0121 (6)	−0.0004 (5)	−0.0013 (5)	−0.0006 (5)
C5	0.0149 (6)	0.0125 (6)	0.0133 (6)	0.0006 (5)	−0.0005 (5)	−0.0014 (5)
C4	0.0165 (6)	0.0196 (7)	0.0196 (7)	−0.0020 (5)	−0.0051 (5)	−0.0009 (5)
C3	0.0193 (6)	0.0165 (6)	0.0148 (6)	0.0005 (5)	−0.0044 (5)	−0.0006 (5)
C6	0.0154 (6)	0.0127 (6)	0.0154 (6)	−0.0035 (5)	−0.0015 (5)	0.0004 (5)
N3	0.0221 (6)	0.0146 (5)	0.0143 (5)	−0.0001 (4)	−0.0021 (4)	0.0007 (4)
N4	0.0262 (6)	0.0161 (6)	0.0162 (6)	0.0006 (5)	−0.0010 (5)	−0.0009 (5)
C10	0.0155 (6)	0.0175 (6)	0.0149 (6)	−0.0022 (5)	−0.0036 (5)	−0.0010 (5)
C9	0.0185 (6)	0.0192 (7)	0.0156 (6)	−0.0020 (5)	−0.0021 (5)	0.0014 (5)
C8	0.0194 (7)	0.0159 (7)	0.0223 (7)	−0.0034 (5)	−0.0057 (5)	0.0022 (5)
C11	0.0277 (8)	0.0199 (7)	0.0161 (6)	0.0016 (6)	−0.0033 (6)	−0.0043 (6)
C12	0.0282 (8)	0.0164 (7)	0.0232 (7)	−0.0002 (6)	−0.0067 (6)	−0.0044 (6)
C7	0.0340 (9)	0.0173 (7)	0.0308 (9)	−0.0031 (6)	−0.0035 (7)	0.0058 (6)
O6	0.0263 (7)	0.0240 (7)	0.0620 (10)	−0.0045 (5)	0.0142 (7)	−0.0127 (7)
O7	0.0273 (7)	0.0304 (7)	0.0498 (9)	−0.0033 (6)	−0.0024 (6)	0.0108 (7)
O8	0.0208 (5)	0.0204 (5)	0.0203 (5)	0.0013 (4)	−0.0044 (4)	−0.0051 (4)

### Geometric parameters (Å, °)

**Table:**

Cu1—O2	1.9644 (13)	N3—C11	1.358 (2)
Cu1—O2^i^	1.9644 (12)	N3—H13	0.8202
Cu1—N1	1.9840 (14)	N4—C10	1.335 (2)
Cu1—N1^i^	1.9840 (14)	N4—H14A	0.7669
Cu1—O5	2.4038 (15)	N4—H14B	0.84 (2)
Cu1—O5^i^	2.4038 (15)	C10—C9	1.412 (2)
O3—C6	1.2467 (18)	C9—C8	1.376 (2)
O4—C6	1.2597 (18)	C9—H9	0.95 (2)
O1—C1	1.2364 (18)	C8—C12	1.416 (2)
O5—H5B	0.79 (3)	C8—C7	1.500 (2)
O5—H5A	0.82 (3)	C11—C12	1.356 (2)
O2—C1	1.2732 (18)	C11—H11	0.9300
N1—C3	1.3291 (19)	C12—H12	0.93 (3)
N1—C2	1.3477 (18)	C7—H7A	0.9600
N2—C4	1.333 (2)	C7—H7B	0.9600
N2—C5	1.3486 (18)	C7—H7C	0.9600
C1—C2	1.5119 (19)	O6—H6B	0.81 (4)
C2—C5	1.387 (2)	O6—H6A	0.81 (4)
C5—C6	1.517 (2)	O7—H17A	0.80 (3)
C4—C3	1.393 (2)	O7—H17B	0.76 (4)
C4—H4	0.9300	O8—H8A	0.81 (3)
C3—H3	0.9300	O8—H8B	0.76 (3)
N3—C10	1.3475 (19)		
			
O2—Cu1—O2^i^	180.00 (4)	N1—C3—H3	120.1
O2—Cu1—N1	83.31 (5)	C4—C3—H3	120.1
O2^i^—Cu1—N1	96.69 (5)	O3—C6—O4	126.59 (14)
O2—Cu1—N1^i^	96.69 (5)	O3—C6—C5	116.73 (13)
O2^i^—Cu1—N1^i^	83.31 (5)	O4—C6—C5	116.56 (13)
N1—Cu1—N1^i^	180.00 (7)	C10—N3—C11	122.73 (14)
O2—Cu1—O5	90.70 (5)	C10—N3—H13	116.8
O2^i^—Cu1—O5	89.30 (5)	C11—N3—H13	120.3
N1—Cu1—O5	90.80 (6)	C10—N4—H14A	119.0
N1^i^—Cu1—O5	89.20 (6)	C10—N4—H14B	117.9 (15)
O2—Cu1—O5^i^	89.30 (5)	H14A—N4—H14B	122.6
O2^i^—Cu1—O5^i^	90.70 (5)	N4—C10—N3	118.61 (14)
N1—Cu1—O5^i^	89.20 (6)	N4—C10—C9	123.36 (14)
N1^i^—Cu1—O5^i^	90.80 (6)	N3—C10—C9	118.02 (14)
O5—Cu1—O5^i^	180.00 (5)	C8—C9—C10	120.30 (14)
Cu1—O5—H5B	113 (2)	C8—C9—H9	123.0 (12)
Cu1—O5—H5A	127.8 (19)	C10—C9—H9	116.7 (12)
H5B—O5—H5A	107 (3)	C9—C8—C12	119.11 (14)
C1—O2—Cu1	114.87 (9)	C9—C8—C7	121.06 (15)
C3—N1—C2	119.43 (13)	C12—C8—C7	119.83 (15)
C3—N1—Cu1	129.00 (10)	C12—C11—N3	120.60 (15)
C2—N1—Cu1	111.57 (10)	C12—C11—H11	119.7
C4—N2—C5	117.45 (13)	N3—C11—H11	119.7
O1—C1—O2	126.27 (13)	C11—C12—C8	119.24 (15)
O1—C1—C2	118.40 (13)	C11—C12—H12	117.2 (17)
O2—C1—C2	115.33 (12)	C8—C12—H12	123.5 (17)
N1—C2—C5	120.04 (13)	C8—C7—H7A	109.5
N1—C2—C1	114.86 (12)	C8—C7—H7B	109.5
C5—C2—C1	125.09 (12)	H7A—C7—H7B	109.5
N2—C5—C2	121.23 (13)	C8—C7—H7C	109.5
N2—C5—C6	114.89 (13)	H7A—C7—H7C	109.5
C2—C5—C6	123.87 (13)	H7B—C7—H7C	109.5
N2—C4—C3	122.10 (14)	H6B—O6—H6A	117 (3)
N2—C4—H4	118.9	H17A—O7—H17B	107 (3)
C3—C4—H4	118.9	H8A—O8—H8B	105 (3)
N1—C3—C4	119.74 (14)		

Symmetry codes: (i) −*x*, −*y*, −*z*+1.

### Hydrogen-bond geometry (Å, °)

**Table:**

*D*—H···*A*	*D*—H	H···*A*	*D*···*A*	*D*—H···*A*
N3—H13···O4^ii^	0.82	1.91	2.7221 (19)	169
N4—H14A···O8^iii^	0.77	2.12	2.8879 (19)	177
N4—H14B···O3^ii^	0.84 (2)	2.07 (2)	2.903 (2)	168 (2)
O5—H5B···O7^ii^	0.79 (3)	1.92 (3)	2.703 (2)	173 (3)
O5—H5A···O4^ii^	0.82 (3)	2.09 (3)	2.8556 (18)	157 (3)
O8—H8B···O1^iv^	0.76 (3)	2.03 (3)	2.7838 (18)	173 (3)
O6—H6B···O4^iv^	0.81 (4)	2.06 (4)	2.839 (2)	162 (3)
O7—H17B···O8^v^	0.76 (4)	2.04 (4)	2.797 (2)	172 (4)

Symmetry codes: (ii) *x*, *y*−1, *z*; (iii) −*x*+1, −*y*, −*z*; (iv) *x*+1, *y*, *z*; (v) *x*, *y*+1, *z*.
